# A comparative study of anti-leukemic effects of kaempferol and epigallocatechin-3-gallate (EGCG) on human leukemia HL-60 cells 

**DOI:** 10.22038/AJP.2021.17604

**Published:** 2021

**Authors:** Mohammad Hassan Jokar, Sima Sedighi, Maliheh Moradzadeh

**Affiliations:** 1 *Golestan Rheumatology Research Center, Sayad Shirazi Hospital, Golestan University of Medical Sciences, Gorgan, Iran*; † * Equal first author*

**Keywords:** APL, Kaempferol, EGCG, Apoptosis, MDR

## Abstract

**Objective:**

Acute promyelocytic leukemia (APL) is among the most threatening hematological malignant cancers. Defects in cell growth and apoptotic pathways lead to the pathogenesis of the disease as well as its resistance to therapy; therefore, it is a good model for examining pro-apoptotic agents. The present study compared the molecular mechanism induced by kaempferol and epigallocatechin gallate (EGCG) as well as all-trans retinoic acid (ATRA), in HL-60 leukemia cells during five days.

**Materials and Methods:**

Cell viability was determined by resazurin assay following treatment with ATRA (10 µM), EGCG, and kaempferol (12.5-100 µM), and apoptosis was detected by the ANX V/PI kit. Moreover, the levels of genes involved in apoptosis (*PI3K*, *AKT*, *BCL2*, *BAX*, *P21*, *PTEN*, *CASP3*, *CASP8*, and *CASP9*) and multi-drug resistance (MDR, *ABCB1* and *ABCC1*) were assessed by using real-time PCR test.

**Results:**

Based on the findings, kaempferol decreased cell viability and increased apoptosis in HL60 cells more than EGCG. Apoptosis was induced via extrinsic and intrinsic pathways in HL60 cells by kaempferol and EGCG. In addition, kaempferol and EGCG increased apoptosis and inhibited MDR in a concentration- and time-dependent manner.

**Conclusion:**

Kaempferol at high concentrations can be taken into consideration for treating patients with APL as compared with EGCG.

## Introduction

Acute promyelocytic leukemia (APL) constitutes 10% of AML cases and is a highly threatening hematological malignant cancer formed by chromosomal translocation t (15;17) (q22; q21) (Adams and Nassiri, 2015 ▶). Patients with APL are treated with all-trans retinoic acid (ATRA) that interferes with differentiation or apoptosis (Pitha-Rowe et al., 2003 ▶). Despite significant therapeutic advancements in leukemia, a large number of patients relapse and do not achieve complete remission (Cardinale et al., 2014 ▶). Consequently, the quest for novel anticancer agents has recently received considerable attention. Researchers believe that dietary phytochemical agents may affect chemotherapy and thus assist the treatment of patients with cancer (Hosseini and Ghorbani, 2015 ▶). According to some studies, phytochemicals (such as polyphenolic or flavonoids) isolated from medicinal plants inhibited cell proliferation and caused apoptosis with high efficacy and low toxicity (Shu et al., 2010 ▶, Moradzadeh et al., 2017 ▶). We previously reported that phyto-polyphenols epigallocatechin-3-gallate (EGCG) and kaempferol have pleiotropic effects in the leukemic cell, activate multiple signaling pathways, and thereby affect several aspects of cellular fate such as cell apoptosis, proliferation, and differentiation. Specifically, these natural compounds cause death in tumor cells but not in normal cells (Kashafi et al., 2017 ▶, Moradzadeh et al., 2018 ▶, Moradzadeh et al., 2018 ▶). Green tea is widely consumed in Asia. Epidemiological studies have shown that drinking green tea aids the treatment of different diseases. Based on several *in vivo* and *in vitro* studies, epigallocatechin gallate (EGCG), a major polyphenolic constituent and a potent antioxidant, mediates the biological activity of green tea (Gupta et al., 2003 ▶). The beneﬁcial effects of EGCG have been reported in the treatment of diabetes, cancer, liver diseases, cardiovascular diseases, and neurodegenerative diseases. EGCG also decreases the risk of cancer development in the prostate, bladder, stomach, esophagus, and lung (Yang et al., 2001 ▶; Thielecke and Boschmann, 2009 ▶; Li et al., 2012 ▶; Xiao et al., 2014 ▶; Zhou et al., 2014 ▶). There are reports that EGCG causes cell-cycle arrest in various rat, mouse, and human cell lines. The mechanisms that underlie the anticancer effect of flavonoids are cell proliferation inhibition, antioxidant activity, apoptosis induction, and cell differentiation stimulation (Kanadaswami et al., 2005 ▶). Kaempferol, a widely known flavonol, has been isolated from grapes, strawberries, Brussels sprouts, apples and other fruits and vegetables. Numerous studies have reported that kaempferol induces cell death in various cancer cell lines derived from different tissues (Luo et al., 2010 ▶). It has been shown to decrease the resistance of cancer cells to anti-cancer drugs, including vinblastine and paclitaxel (Limtrakul et al., 2005 ▶). In previous studies, we showed the effects of short-term *in vitro* exposure to EGCG and kaempferol on APL cell lines (Moradzadeh et al., 2018 ▶; Moradzadeh et al., 2018 ▶). The present study thus aimed to compare the molecular mechanism of kaempferol and EGCG against leukemia HL-60 cell line in a period of five days. To this end, a series of experiments were designed in order to investigate the impacts of kaempferol and EGCG on proliferation, apoptosis, and multi-drug resistance in leukemia HL60 cell line.

## Materials and Methods

**Chemicals and reagents**

High-glucose Roswell Park Memorial Institute (RPMI 1640) medium and fetal bovine serum (FBS) were procured from Gibco (USA). TRIzol was provided from Invitrogen (USA). Penicillin-streptomycin solution, all-trans retinoic acid (ATRA), kaempferol (>97%), EGCG (>95%), and 7-hydroxy-3H-phenoxazin-3-one-10-oxide (resazurin) were purchased from Sigma-Aldrich (USA). FITC annexin V kit was obtained from BD Biosciences (USA). Human promyelocytic leukemia HL60 cell line was procured from the cell bank of Pasteur Institute (Iran). The Real-time PCR Master Mix and cDNA synthesis Kit were purchased from Roche Diagnostic (Switzerland) and Fermentas (Lithuania), in respective order.

**Cell proliferation assay**

Cell proliferation was examined via resazurin reduction by live cells to resorufin, a highly fluorescent compound. After treating HL60 cells with kaempferol (12.5-100 µM) and EGCG (12.5-100 µM) or ATRA (10 µM), 20 µl of the resazurin reagent [300 μM resazurin, 78 μM methylene blue, 1 mM potassium hexacyanoferrate III, and 1 mM potassium hexacyanoferrate II] was added to each well. After 4 hr, fluorescence intensity was measured by a fluorescence Victor X5 2030 Multilabel Plate Reader (PerkinElmer, Shelton, Connecticut) at an an emission wavelength of 590 nm and an excitation wavelength of 530 nm (Mashkani et al., 2016 ▶).

**Apoptosis assay**

The impacts of kaempferol and EGCG on the apoptosis of HL60 cells were assessed by the ANX V/PI staining method. The cells were treated with kaempferol and EGCG (100 µM) or ATRA (10 µM), and subsequently incubated with the ANX V/PI reagent. The quantification of apoptosis was performed by FACS Calibur (BD Biosciences) flow cytometer followed by analysis using Flowjo software (TreeStar, Inc. Country) (Rangarajan et al., 2015 ▶). 

**Real-time PCR quantification using SYBR Green**

The HL60 cells treated with kaempferol and EGCG (100 µM) or ATRA (10 µM) were subjected to RNA extraction using TRIzol according to the manufacturer’s instruction. The RNA concentration and purity were determined by spectrophotometry. cDNA was synthesized from the total RNA (100 ng) of each sample using a cDNA synthesis kit using random hexamer primers. Primers were designed in Beacon software (Applied Biosystems, USA) (Moradzadeh et al., 2018 ▶). Gene expression changes were measured for genes of ATP-binding cassette (ABC) membrane transporters and apoptosis pathways (*PI3K*, *AKT*, *BCL2*, *BAX*, *p21*, *PTEN*, *CASP3*, *CASP8* and *CASP9*) using SYBR Green-based real-time PCR technology by Applied Biosystems Step One plus detection system (ABI, USA). The reaction mixture contained 2 μl of cDNA (250-400 ng), 1 μl of the primers (100 pmol), 10 μl of 2 x SYBR Green master mixes, and dH_2_O to bring the volume to 20 μl. The optimized parameters utilized for the thermocycler were short hot-start at 95°C for 15 min, followed by 40 cycles, each comprising denaturing at 95°C for 15 sec, annealing at 60°C for 1 min, and extension at 72°C for 20 sec. As the last step of SYBR Green real-time PCR, melting curves were incorporated from 60 to 90°C, rising by 0.3 degrees. The samples were run in triplicate and the fold difference of expression in the treated and untreated samples was calculated using the 2^-ΔΔCt^ method (Pfaffl, 2006). The expressions of genes were normalized to *GAPDH* and *β-actin* genes. 

**Statistical analysis**

The data are presented as mean±SEM and were analyzed by one-way analysis of variance (ANOVA) and Tukey’s multiple-comparison test. The p-value<0.05 was considered statistically significant. Statistical analysis was performed in Graph Pad PRISM software (Version 6, Graph Pad Software, CA).

## Results

**Kaempferol and EGCG inhibited the proliferation of leukemia HL60 cells**

**Figure 1:**
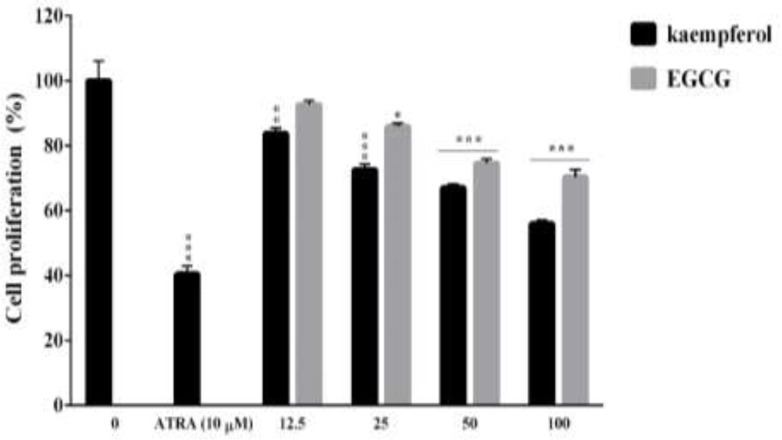
The effects of kaempferol and EGCG on the proliferation of leukemia HL60 cells. The cells were treated up to five days with different concentrations of kaempferol and EGCG (12.5-100 µM) or all-trans retinoic acid (ATRA). Cell proliferation was examined by the resazurin assay. The data are expressed as the mean±SEM of three independent experiments performed in triplicate. *p<0.05, **p<0.01, and ***p<0.001 vs. the corresponding untreated control cells (concentration of 0).

Kaempferol at concentrations of 25-100 μM and EGCG at concentrations of 50 and 100 μM significantly reduced the proliferation of HL60 cells on day 5 (p<0.001) (Figure 1). Similarly, a signiﬁcant decrease in proliferation was seen in cells treated with 10 μM of ATRA (p<0.001). The IC_50_ values of kaempferol and EGCG were 99.28±0.03 and 190.4±0.03 μM in HL60 cells after 5 days of incubation, respectively. In previous studies, we showed that kaempferol and EGCG did not affect the proliferation of normal polymorph nuclear cells (Moradzadeh et al., 2018 ▶, Moradzadeh et al., 2018 ▶). 

**Figure 2:**
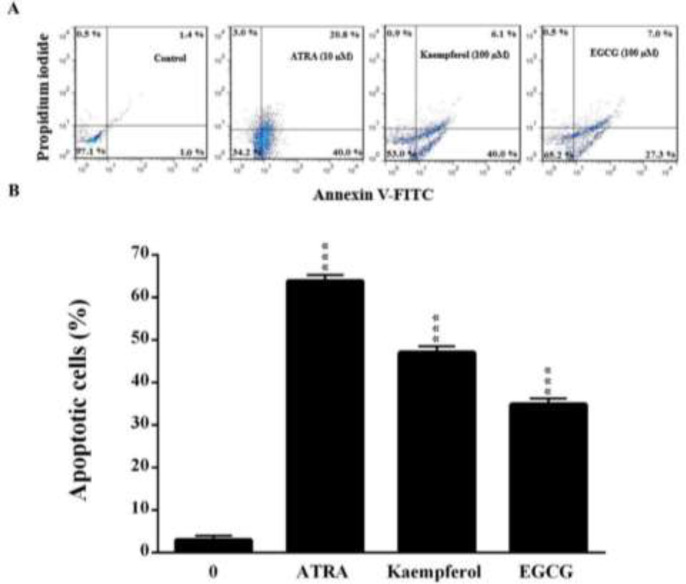
The effects of kaempferol and EGCG on apoptosis in leukemia HL60 cells as evaluated by annexin V and propidium iodide double-staining. The cells were treated for five days with kaempferol and EGCG (100 µM) or ATRA. (A) A representative histogram of the fluorescence intensity of annexin V and PI double-stained cells (x-axis: green fluorescence of annexin-V-FITC indicating apoptotic cells; y-axis: red fluorescence of PI depicting necrotic cells). (B) Quantitative analysis of apoptosis as demonstrated in (A). The data are expressed as the mean±SEM of three independent experiments performed in triplicate. ***p<0.001 vs. the untreated control cells (concentration of 0).

**Kaempferol and EGCG enhanced apoptosis in leukemia HL60 cells**

Figure 2 illustrates the effects of kaempferol and EGCG on apoptosis in leukemic cells as evaluated by ANX V and PI double staining. Like ATRA (10 µM), kaempferol and EGCG at a concentration of 100 µM significantly increased the apoptosis rate of HL60 cells after 5 days (p<0.001).

**Kaempferol and EGCG modulated genes involved in survival and apoptosis in leukemia HL60 cells**

**Figure 3:**
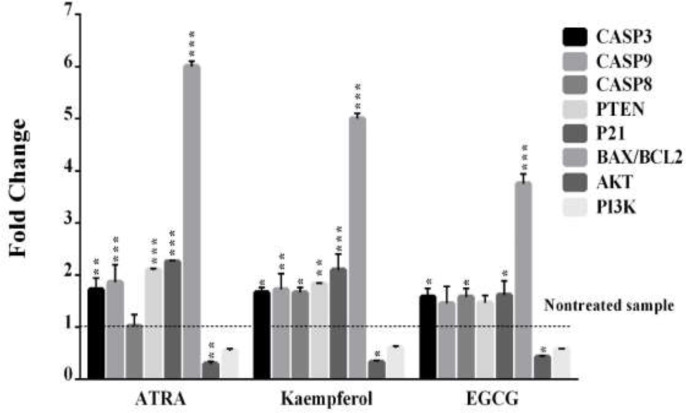
The effects of kaempferol and EGCG on the expression of apoptotic and anti-apoptotic genes in leukemia HL60 cells. The cells were treated for five days with kaempferol, EGCG, or ATRA, and the expression of apoptotic (*CASP3, CASP9, CASP8, P21, PTEN *and* Bax*) and anti-apoptotic (*PI3K, AKT *and* Bcl2*) genes was checked by real-time PCR. The data are expressed as the mean±SEM of three independent experiments performed in triplicate. *p<0.05, **p<0.01, and ***p<0.001 vs. the untreated control cells

Figure 3 depicts the effects of 5 days of incubation with kaempferol and EGCG on the expression of genes involved in survival (*PI3K*, *AKT* and *Bcl2*) and apoptosis (*CASP3*, *CASP9*, *CASP8*, *P21*, *PTEN* and *Bax*). In HL60 cells, kaempferol significantly increased the expressions of *CASP3*, *CASP9*, *CASP8*, *PTEN* and *P21* but decreased the expression of *AKT* (p<0.05). Similarly, EGCG significantly increased *CASP8*, *CASP3* and *P21* but reduced *AKT *expression in the cells (p<0.05). Considering the gene expression of *Bax*/*Bcl-2*, kaempferol and EGCG (100 μM) significantly increased 5 and 3.7-fold vise 6-fold in ATRA (10 μM)–treated HL60 cells after 5 days (p<0.001).

**Kaempferol and EGCG decreased the expression of MDR genes in leukemia HL60 cells**

**Figure 4:**
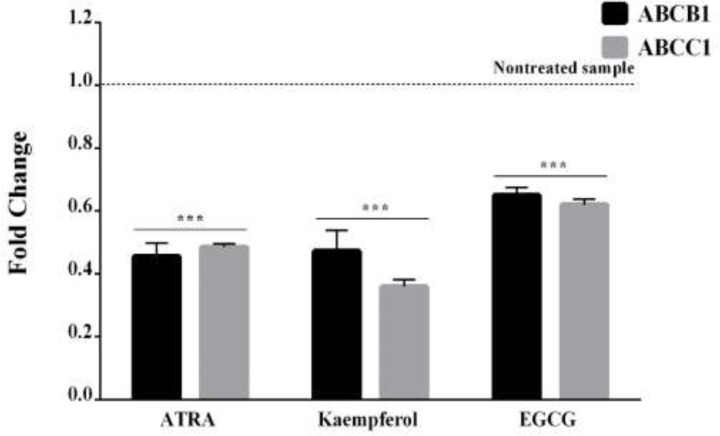
The effects of kaempferol and EGCG on the expression of ATP-binding cassette (ABC) membrane transporter genes in leukemia HL60 cells. The cells were treated for five days with kaempferol and EGCG or ATRA, and the expressions of *ABCB1* and *ABCC1* genes were investigated by real-time PCR. The results are the mean±SEM of three different experiments. ***p<0.001 vs. the untreated control cells

Based on the real-time PCR assay, 5 days of incubation with ATRA (10 µM), kaempferol, and EGCG (100 µM) decreased the expressions of *ABCB1* and *ABCC1* genes in leukemia HL60 cells (Figure 4, p<0.001).

## Discussion

This was the first study to simultaneously explore the mechanism of apoptotic cell death and multi-drug resistance induced by kaempferol and EGCG in HL-60 leukemic cells compared with a standard anti-promyelocytic leukemia drug, ATRA, in the long term (Moradzadeh et al., 2017 ▶). We had reported in previous studies that EGCG and kaempferol inhibit cell growth, increase apoptosis and induce differentiation in leukemia NB4 and HL60 cells within short-term exposure. The cytotoxic impact of kaempferol was more prominent against leukemic cells than normal human PMN cells, and these effects were comparable with those of ATRA (Moradzadeh et al., 2018 ▶; Moradzadeh et al., 2018 ▶). Our data indicated that kaempferol compared to EGCG, further inhibited cell growth and induced apoptosis in leukemia HL60 cells by inhibiting MDR and increasing Bax/Bcl2. As compared to three-day treatment, the IC50 of kaempferol was significantly reduced, whereas the IC50 of EGCG increased after five days. Based on the results of ANX V/PI staining, like ATRA, kaempferol enhanced apoptosis in the leukemia cells more than EGCG after five-day incubation. It managed to reduce the proliferation rate of APL cells, and this effect is consistent with those reported previously on the inhibitory characteristic of kaempferol against different cancerous cells (Xu et al., 2008 ▶; Luo et al., 2010 ▶; Luo et al., 2011 ▶; Alonso-Castro et al., 2013 ▶; Tu et al., 2016 ▶; Da et al., 2019 ▶; El-Kott et al., 2020 ▶). In previous studies, we showed that EGCG and kaempferol increased apoptosis and inhibited telomerase in breast and cervical cancer cells (Kashafi et al., 2017 ▶; Moradzadeh et al., 2017 ▶). In contrast, Kang et al. showed that kaempferol did not affect F9 cells growth (Kang et al., 2011 ▶). Another study concluded that kaempferol shows antitumor characteristics *in vitro* and *in vivo* in malignant glioma models (Santos et al., 2015 ▶). Other studies have also shown the inhibitory property of EGCG against different cancerous cells (Benyahia et al., 2004 ▶; Khan et al., 2010 ▶; Ly et al., 2013 ▶; Wang et al., 2015 ▶). Apoptosis starts by two main signaling pathways, known as extrinsic and intrinsic pathways. The former, CASP8, is activated, thereby inducing apoptosis by stimulating CASP3. The latter pathway stimulates a CASP9/3 signaling cascade leading to DNA fragmentation and apoptosis. The ratios of proapoptotic and antiapoptotic proteins of the BCL2 family, and more importantly, the BAX/BCL2 ratio, control the resistance or sensitivity of cells to apoptotic stimuli (Ouyang et al., 2012 ▶). The activation of pro-survival pathways, such as PI3K/AKT signaling cascade, helps cells to resist apoptosis triggers. Some tumor suppressors such as PTEN block the PI3K/AKT signaling pathway, thereby inhibiting cell growth and apoptosis (Ouyang et al., 2012 ▶). In the present study, kaempferol and EGCG significantly up-regulated pro-apoptotic genes (*CASP3*, *CASP8*, *P21* and *BAX*) but reduced the expressions of survival genes (*AKT* and *Bcl-2*) in leukemic cells. Moreover, CASP8 and the ratio of *Bax*/*Bcl-2* significantly increased in kaempferol- and EGCG-treated cells, demonstrating that kaempferol and EGCG induced apoptosis in leukemic cells through both intrinsic and extrinsic pathways. Compared to the three-day period, the Bax/Bcl2 ratio in kaempferol-treated HL60 cells was more than EGCG following five-day incubation. Leukemic cells usually express high levels of MDR-associated proteins such as ABCB1 and ABCC1, posing a challenge to successful treatment of some patients with ATRA (Pallis et al., 2002 ▶; Shaffer et al., 2012 ▶). Here, we also showed that, as compared to EGCG, kaempferol at high concentrations can further decrease the expression of *ABCB1* and *ABCC1* in HL60 cells in the long term (5 days), an effect which is consistent with some studies reporting that kaempferol inhibited MDR proteins in some cancer cells (Kitagawa et al., 2005 ▶; Limtrakul et al., 2005 ▶; Nakamura et al., 2005 ▶; Pal and Mitra, 2006 ▶; Chung et al., 2007 ▶; To et al., 2012 ▶; Kuete et al., 2014 ▶). According to a study, kaempferol was ineffective in P-gp inhibition on cancer cell lines (Gyemant et al., 2005 ▶). Also, EGCG decreased MDR in various cancer cells (Lyn-Cook et al., 1999 ▶; Mei et al., 2005 ▶; Qian et al., 2005 ▶; Nowakowska and Tarasiuk, 2016 ▶; Wen et al., 2017 ▶), and this supports the view that EGCG might suppress drug resistance via the downregulation of ABC transporters. 

In conclusion, the results of the present study demonstrated that, in comparison to EGCG, kaempferol has larger antiproliferative and proapoptotic impacts on human leukemia HL60 cells by increasing *CASP8* and intracellular *BAX*/*BCL2* ratio and reducing the expressions of MDR-associated genes in the long term. Based on these findings, kaempferol at high concentrations, might be more efficient than EGCG for treatment of patients with APL.
